# Spontaneous Gelation of Adhesive Catechol Modified Hyaluronic Acid and Chitosan

**DOI:** 10.3390/polym14061209

**Published:** 2022-03-17

**Authors:** Guillermo Conejo-Cuevas, Leire Ruiz-Rubio, Virginia Sáez-Martínez, Raul Pérez-González, Oihane Gartziandia, Amaia Huguet-Casquero, Leyre Pérez-Álvarez

**Affiliations:** 1 Macromolecular Chemistry Group (LABQUIMAC), Department of Physical Chemistry, Faculty of Science and Technology, University of the Basque Country, UPV/EHU, Barrio Sarriena, s/n, 48940 Leioa, Spain; gconejo001@ikasle.ehu.eus (G.C.-C.); leire.ruiz@ehu.eus (L.R.-R.); 2 BCMaterials, Basque Center for Materials, Applications and Nanostructures, UPV/EHU Science Park, 48940 Leioa, Spain; 3 i+Med S. Coop. Parque Tecnológico de Álava, Albert Einstein 15, nave 15, 01510 Vitoria-Gasteiz, Spain; v.saez@imasmed.com (V.S.-M.); r.perez@imasmed.com (R.P.-G.); o.gartziandia@imasmed.com (O.G.); amaya.huguet@imasmed.com (A.H.-C.)

**Keywords:** hyaluronic acid, chitosan, catechol, tissue adhesive

## Abstract

Spontaneously formed hydrogels are attracting increasing interest as injectable or wound dressing materials because they do not require additional reactions or toxic crosslinking reagents. Highly valuable properties such as low viscosity before external application, adequate filmogenic capacity, rapid gelation and tissue adhesion are required in order to use them for those therapeutic applications. In addition, biocompatibility and biodegradability are also mandatory. Accordingly, biopolymers, such as hyaluronic acid (HA) and chitosan (CHI), that have shown great potential for wound healing applications are excellent candidates due to their unique physiochemical and biological properties, such as moisturizing and antimicrobial ability, respectively. In this study, both biopolymers were modified by covalent anchoring of catechol groups, and the obtained hydrogels were characterized by studying, in particular, their tissue adhesiveness and film forming capacity for potential skin wound healing applications. Tissue adhesiveness was related to o-quinone formation over time and monitored by visible spectroscopy. Consequently, an opposite effect was observed for both polysaccharides. As gelation advances for HA-CA, it becomes more adhesive, while competitive reactions of quinone in CHI-CA slow down tissue adhesiveness and induce a detriment of the filmogenic properties.

## 1. Introduction

Hydrogels are polymers based on three-dimensional networks capable of retaining large amounts of water due to their hydrophilic nature, while remaining insoluble due to polymer chains crosslinking [1]. Crosslinking by covalent bonds results in covalently crosslinked networks, and when polymers are joined by non-covalent interactions such as hydrogen bonds and hydrophobic or dipole–dipole interactions, physical hydrogels are formed [2]. Dried hydrogels behave similarly to a hard solid, but in an aqueous medium, water penetration between the polymer chains causes the swelling of the network [3,4]. Water content affects dramatically the mechanical properties of these gels [1], leading to soft, elastic and permeable materials for which its properties are similar to those of biological tissues [5]. For this reason, hydrogels are well known as interesting materials in biomedical applications [6].

Hydrogel characteristics make them interesting candidates for wound healing applications. On the one hand, their hydrophilic nature allows the required moist environment in the wound for extracellular matrix formation and re-epithelialization and provides protection against infections. On the other hand, the incorporation of therapeutic agents into hydrogels acting as wound dressings provides their topical release in the wound that has been shown to be more effective than systemic treatment [7]. Indeed, the promotion of an effective wound healing or regeneration, which consists of a series of complex biochemical reactions that aims to repair the wound, is highly demanded. This process occurs in three stages that can take place simultaneously. Firstly, the inflammation phase takes place, which can be summarized as the elimination of bacteria and the migration of cells that act in the second stage. Secondly, the proliferation phase comprises an increase in collagen with the aim of forming new tissues and blood vessels, as well as the contraction of the wound. Lastly, in the last phase called maturation, the elimination of the excess cells and the repositioning of the collagen occur. This entire process is complex and highly susceptible to be interrupted or fail [8]. Due to the latter, this process can be supported by healing species, which can help by functioning as antibacterial barriers or by acting as cellular scaffolds that enhance wound closure [6].

Hydrophilic polymers, due to their ability to mimic physical and biological properties of tissues, can promote damaged tissue regeneration. In this sense, it is worth highlighting that hydrogels are derived from natural polymers, especially polysaccharides, which have been widely investigated and exploited in recent years due to their abundance, biocompatible, filmogenic, and beneficial biological properties that make them interesting candidates for wound dressing applications [2]. The most studied polysaccharides include alginates, chondroitin, chitosan and chitin, cellulose, dextran, hyaluronic acid and heparin.

**Figure 1:**
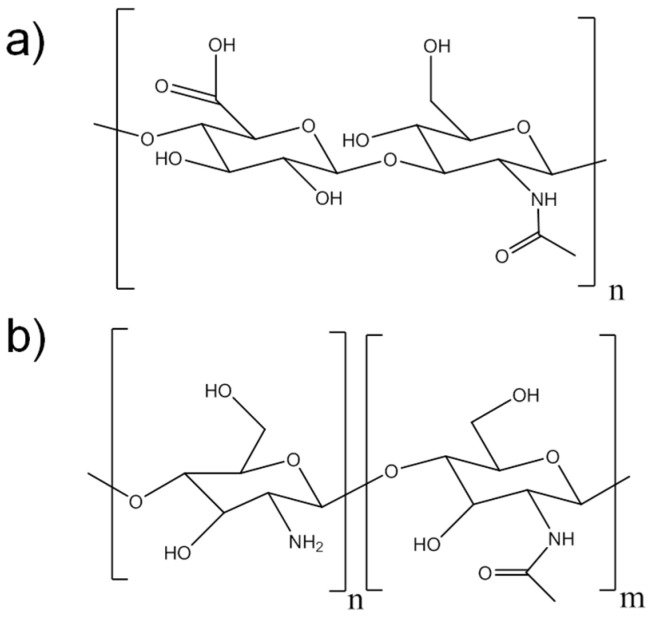
(**a**) Molecular structure of hyaluronic acid showing *D*-glucuronic acid (left) and a *N*-acetyl-*D*-glucosamine (right) units. (**b**) Molecular structure of chitosan monomers possessing *D*-glucosamine (left) and *N*-acetylglucosamine (right).

Hyaluronic acid (HA) is a natural polysaccharide based on a *D*-glucuronic acid and *N*-acetyl-*D*-glucosamine (Figure 1b) that is used in various biomedical applications, such as wound healing, visco-supplementation for wrinkle fillers, drug delivery carriers and tissue scaffolds. Furthermore, this polysaccharide is completely degraded in the body by hyaluronidase, in which the velocity of biodegradation is influenced by its molecular weight [9]. HA can interact intra/intermolecularly thanks to hydrogen bonds or ionic interactions by its carboxylic groups and their deprotonated form, carboxylates. It also can be easily modified by its carboxyl or hydroxyl groups. There are many examples of HA modified hydrogels for healing, such as hyaluronic acid modified with bromo acetate or those modified with polyhydrazides [10].

Chitosan (CHI) is also a natural polymer that comes from the partial deacetylation of chitin, a natural polymer synthesized by some arthropods, fungi and insects [11,12]. Thus, chitosan has a *D*-glucosamine structure mixed with *N*-acetylglucosamine structures for the acetylated monomer, as observed in Figure 1a. Chitosan is degraded in the human body by the action of lysozyme and colonic bacterial enzymes and its biodegradation strongly depends on its deacetylation degree and molecular weight [2]. It is considered one of the most promising materials in the fields of pharmacy, chemistry and the food industry due to its highly reactive hydroxyl and amino groups, as well as being a biocompatible, antibacterial and nontoxic polymer [13]. It is also noteworthy its ability to form films, which it is able to cause the suppression of essential nutrients for microbial growth, in other words protecting the open wound from the outside due to its good barrier properties [14].

Due to the presence of cited chemical groups in its structure, CHI is very good at interacting through hydrogen bonds, and by electrostatic interactions with negative charges at the appropriate pH due to the protonation of its amino groups (-NH_3_^+^) [2].

Since wound healing treatment requires prolonged time periods, the development of filmogenic materials with tissue adhesiveness, such as adhesive hydrogels, is crucial for a suitable performance on the skin. For this, acrylate derived hydrogels have been typically developed in the last decades based on their adhesive properties [11,15].

**Figure 2:**
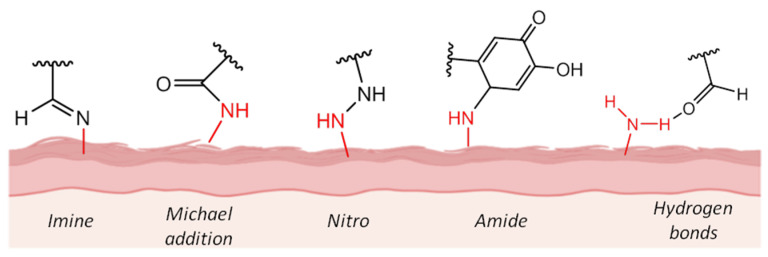
Intermolecular interactions between polymer chains and tissue.

Tissue adhesion (Figure 2) is promoted by the interaction of tissues with many functional groups that are present along hydrogels polymeric chains through covalent bonds, such as imine formations or Schiff bases and Michael additions, among others. Moreover, tissue adhesion promoted by physical interactions such as hydrogen bonding is the most frequent. However, these interactions are reversible, which causes a decrease in the ability of the hydrogel to remain attached to tissues [16].

In nature, the adhesive ability of mussels has been ascribed to the presence of an amino acid: L-3,4-dihydroxyphenylalanine (DOPA), which is responsible of their adhesion to both inorganic and organic surfaces, especially in humid conditions. This dihydroxy group is called catechol (CA) [17,18].

**Figure 3:**
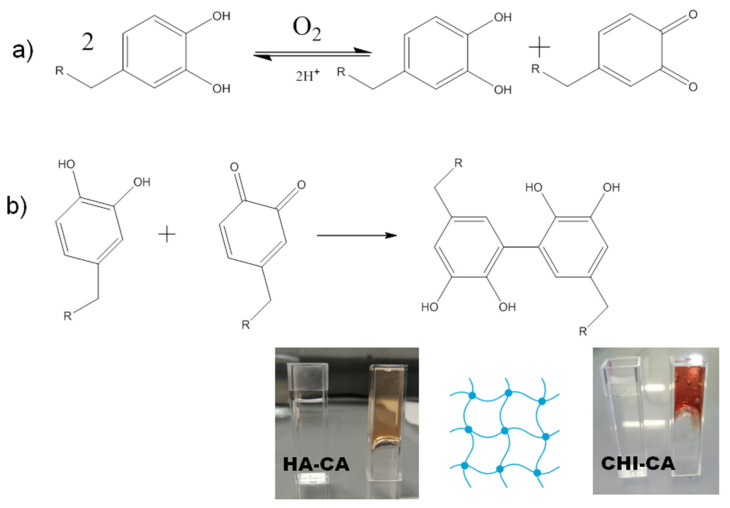
(**a**) Catechol group oxidation to quinone by atmospheric oxygen. (**b**) Crosslinking between catechol and quinone groups. R equals to CHI or HA.

Taking the inspiration of these natural organisms, the strategy of modifying polymers with catechol groups has recently been developed to discover new materials, such as hydrogels with adhesive properties [12]. The derivatives of catechol are particularly interesting, as they are also natural and, therefore, biodegradable and biocompatible. The main advantage of natural polysaccharides chitosan and hyaluronic acid relies on the fact that they are easily modifiable through chemical reactions by their amine or carboxylic acid groups, respectively [9,19]. Moreover, the CA group can be oxidized at basic pH or even in the presence of the oxygen of the atmosphere [9], and it is transformed to o-quinone (Figure 3a). It can also be oxidized intentionally and at a higher rate with sodium periodate [20]. This spontaneously formed group behaves as a Michael acceptor and reacts with specific substrates, such as amines, thiols, alcohols, etc. [21,22].

**Figure 4:**
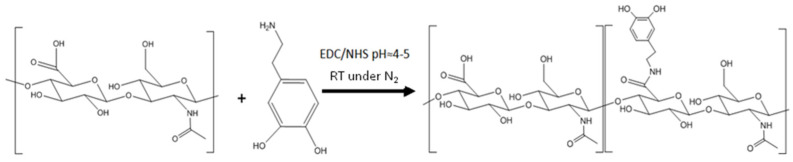
HA-CA synthesis reaction at pH = 4–5 at room temperature and under nitrogen atmosphere.

In the case of organic surfaces that contain electron donor groups such as alcohols, thiols or amines, quinone reacts irreversibly, making more resistant covalent bonds than physical interactions of catechol [23]. Once CA is oxidized to quinone, the polymer that carries this substituent begins to react with itself (Figure 4) [22,23], causing its self-crosslinking, increasing viscosity, while a change of colour takes places and becomes brownish. This reaction results in a rapid hardening of the product (Figure 3b) that can be seen as an advantage, because it is a method for spontaneously promoting the gelation of the polymer, which proceeds from a viscous liquid state to a gelled state without the addition of any external crosslinking agents. However, to the best of our knowledge, the effect of this spontaneous gelation on the tissue adhesiveness and filmogenic properties of these polysaccharides has not been explored and comparatively analyzed.

Taking all this into account, this work aims to explore the formation of hydrogels of catechol derivatives obtained by the chemical modification of chitosan and hyaluronic acid as tissue adhesive and filmogenic materials for potential wound healing purposes.

## 2. Materials and Methods

### 2.1. Materials

For the synthesis of the hydrogels, chitosan (CHI, 1.2 × 10^6^ ± 153.9 g/mol, Sigma-Aldrich, St. Louis, MO, USA; DD = 80%), hyaluronic acid (HA, 1.9–2.2 × 10^6^ g/mol, Contripo, Dolní Dobrouč, Czech Republic), hydrochloric acid (HCl, 37%, Panreac, Barcelona, Spain) and ethanol (EtOH, 99.8%, Panreac, Barcelona, Spain) as solvent were used. 3,4-Dihydroxycinnamic acid or hydrocaffeic acid (HCF, 98%, Sigma-Aldrich, St. Louis, MO, USA) and dopamine hydrochloride (DOPA, 98%, Sigma-Aldrich, St. Louis, MO, USA) were employed to introduce the catechol group. To carry out the conjugation of the catechol to the polymer, N-hydroxysuccinimide (NHS, 98%, Sigma-Aldrich), St. Louis, MO, USA and N-(3-Dimethylaminopropyl)-N′-ethylcarbodiimide hydrochloride (EDC, 98%, Sigma-Aldrich, St. Louis, MO, USA) were used. Subsequently, to clean the modified polymer, a dialysis was carried out with 12,000 Da membranes (Medicell Membranes Ltd., London, UK). The magnesium chloride salt (MgCl_2_, 98%, Sigma-Aldrich) was used to control humidity in a closed atmosphere. Sodium metaperiodate (NaIO_4_, 99%, Sigma-Aldrich, St. Louis, MO, USA) was used for catechol oxidation in spectroscopy calibration. In order to prepare a phosphate buffer saline or PBS, monobasic sodium phosphate (NaH_2_PO_4_, 99%, Sigma-Aldrich, St. Louis, MO, USA) and sodium hydroxide (NaOH, 99%, Panreac) were used. PET films (75 μm) were supplied by HIFI Film Industria (Stevenage, UK).

### 2.2. Experimental Synthesis

#### 2.2.1. Synthesis of Hyaluronic Acid-Catechol (HA-CA)

The synthesis of hyaluronic acid with catechol was carried out following the described method [24]. Briefly, high molecular weight hyaluronic acid (1 g, 2.5 mmol) was dissolved in distilled water (200 mL) for 12 h and under a nitrogen atmosphere. EDC (959 mg and 5 mmol) and NHS (575 mg and 5 mmol) (Figure 4) were then slowly added to the reaction flask. After 20 min under stirring, dopamine hydrochloride (948 mg, 5 mmol) was added at pH 4–5 for 4 h. It was left to react overnight and dialyzed in 12,000–14,000 Da dyalisis membranes against acidified deionized water (pH 5) for 3 days. Finally, the product was lyophilized and stored in a vacuum desiccator at 3 °C.

#### 2.2.2. Chitosan-Catechol Synthesis

**Figure 5:**
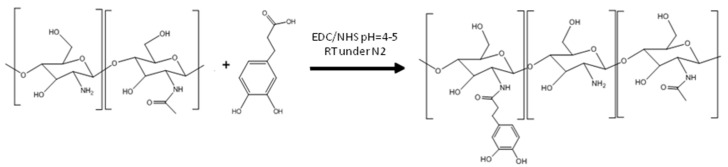
CHI-CA synthesis reaction at pH = 4–5 at room temperature under nitrogen atmosphere.

The synthesis of chitosan modified with the catechol group was carried out following the described method [17]. Briefly, high molecular weight chitosan (591 mg and 1.6 mmol) was dissolved in 22.5 mL of water together with 2.5 mL of 1 M HCl overnight under a nitrogen atmosphere. The next day, hydrocaffeic acid (600 mg and 3.25 mmol), previously dissolved in 1.5 mL in distilled water, was added. Then, EDC (930 mg and 4.75 mmol) and NHS (558 mg and 4.75 mmol) (Figure 5), dissolved in 50 mL of an ethanol/water solution (1:1, *v*/*v*), were added. The reaction was left overnight, and the pH value was between 4 and 5. The product was dialyzed on 12,000–14,000 Da membranes in acidified deionized water (pH 5) for 3 days. Finally, the product was lyophilized (Benchtop Freeze Dryer operating at −50 °C, 0.1 mBar) and stored in a vacuum desiccator at 3 °C.

#### 2.2.3. Films

HA-CA and CHI-CA films were prepared by using a doctor blade technique to form wet films with well-defined thicknesses from solutions at a concentration of 7 g/L in water at room temperature. Films that were 1-millimeter-thick were obtained onto the PET sheet.

### 2.3. Characterization Techniques

Proton nuclear magnetic resonance (^1^H NMR) spectra were performed at room temperature on a Bruker AV-500 spectrometer (500 MHz for 1H), using deuterated acetic acid and water as solvents. Chemical shifts (δ) are expressed in parts per million with respect to deuterated water. The concentration of quinone group was determined by ultraviolet and visible spectroscopy (UV-VIS) measuring the absorbance at 414 nm, respectively, in the Double beam Cintra303 GBC equipment. The gelation time of prepared hydrogels was determined at different polysaccharide concentrations by the known inverted tube test [9], in which it is considered that the gelation point corresponds to the moment in which the solution stops flowing once inverting the tube. An inverted optical microscope Olympus IX71 from Japan was used as a non-destructive technique. Photographs were obtained in order to study the stability of the films during and after drying. A Hitachi S-4800 brand scanning electron microscope (FEG-SEM) from Japan was used in order to obtain high resolution images of the films at micron scale. In the case of polymers, a layer of gold was applied to allow the mobility of the electrons because they are not conductive. The adhesion of the synthesized hydrogels was determined by measuring the force necessary to detach gels from a piece of tissue with mechanical test equipment (Metrotec, MTEf), using a 20 N load cell. For this purpose, porcine skin without external fat was cut into circular sections of 196 mm^2^ and kept for 4–5 h in a PBS solution (pH ≈ 7.4) at 37 °C to simulate physiological conditions [15,25]. Then, skin was fixed with cyanoacrylate (Loctite^®^) [26] to a test tube and placed on the surface of the gel sample. Finally, the force per area required to detach it from the sample was measured. The stress–displacement curves were obtained for each sample. All measurements were conducted with the following parameters: test speed: 2 mm/min; skin/sample contact time: 1 min; contact area: 196 mm^2^; preload: 0 N; drop: 100%.

## 3. Results

### 3.1. Catechol Conjugation

**Figure 6:**
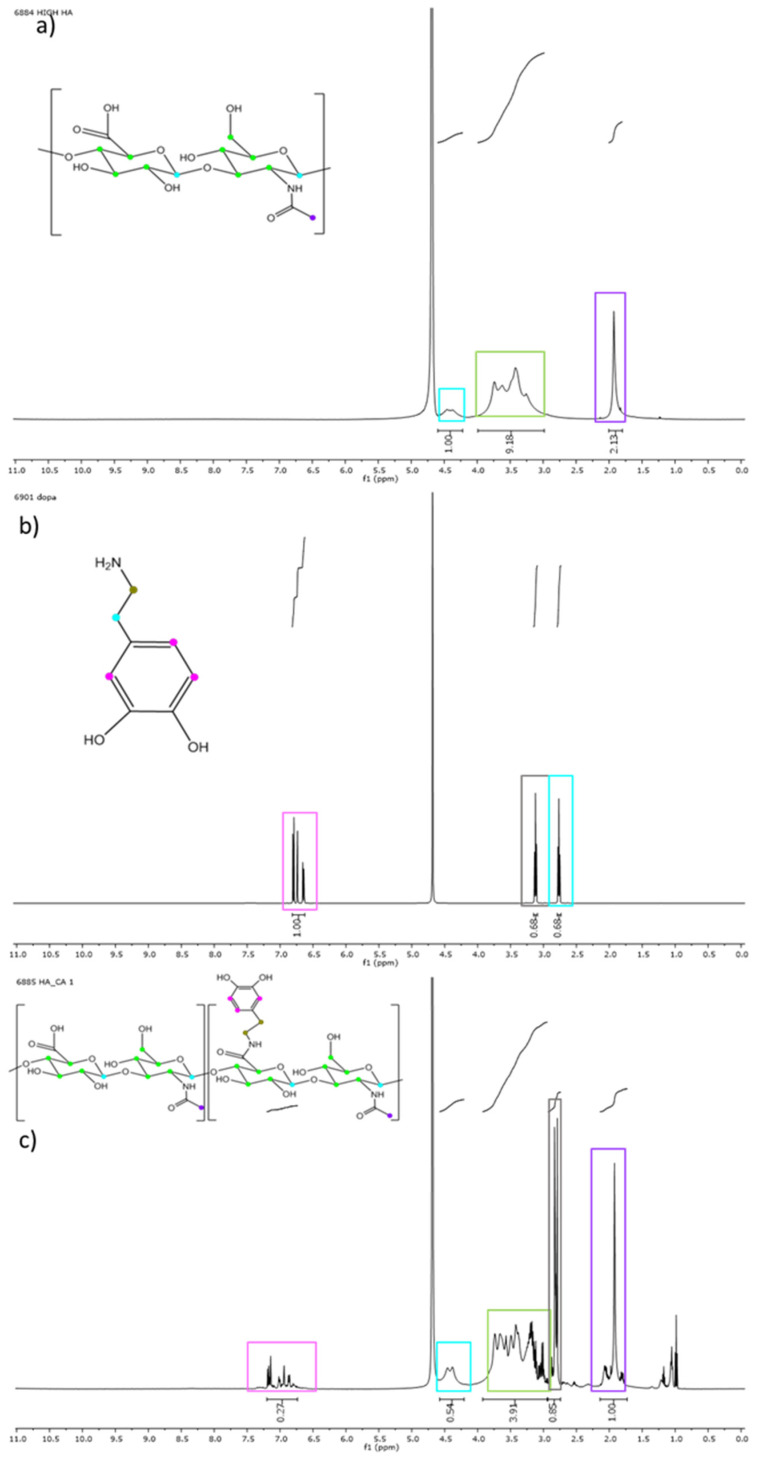
(**a**) High molecular weight hyaluronic acid spectrum. (**b**) Dopamine hydrochloride spectrum, a reagent that adds catechol to the product. (**c**) Hyaluronic acid modified with catechol (HA-CA) spectrum.

CHI and HA were chemically modified, as described in the Experimental Section, in order to introduce catechol groups along polysaccharides chains to promote gelation and enhance adhesiveness to biological tissue. This conjugation was confirmed and quantified by ^1^H NMR and UV analyses. Figure 6 compares the ^1^H-NMR spectra of initial HA, dopamine hydrochloride reagent and the finally modified HA-CA.

^1^H-NMR Hyaluronic acid (D_2_O, 500 MHz, 20 °C): δ (ppm) = 4.30 (s, 2H, anomeric CH), 3.00–4.00 (m, 10H, ring CH and CH_2_), 1.99 (s, 3H, acetamide).

^1^H-NMR Dopamine Hydrochloride (D_2_O, 500 MHz, 20 °C): δ (ppm) = 6.70 (m, 3H, CH aromatic ring), 3.15 (d, 2H, -CH_2_N), 2.75 (d, 2H, -CH_2_Ar).

^1^H-NMR Hyaluronic acid-catechol (D_2_O, 500 MHz, 20 °C): δ (ppm) = 6.75–7.30 (m, 3H, CH aromatic ring), 4.30 (s, 4H, anomeric CH), 3.00–4.00 (m, 20H, ring CH and CH_2_), 2.82 (d, 2H, CH_2_N), 2.80 (d, 2H, -CH_2_Ar), 1.99 (s, 6H, acetamide).

The appearance of new peaks corresponding to the phenyl hydrogens of catechol moieties (Figure 6c) at 6.5–6.75 ppm and those appearing at 2.75 ppm ascribed to the aliphatic carbons of catechol [9] demonstrates the successful conjugation of HA with catechol functionality. In addition, the integration of the peaks at 6.5–6.75 ppm with respect to 1.99 ppm peak corresponding to the methyl protons of the acetamide group of HA allows the quantification of the percentage of introduced catechol groups, obtaining average substitution values of 38 ± 8%.

**Figure 7:**
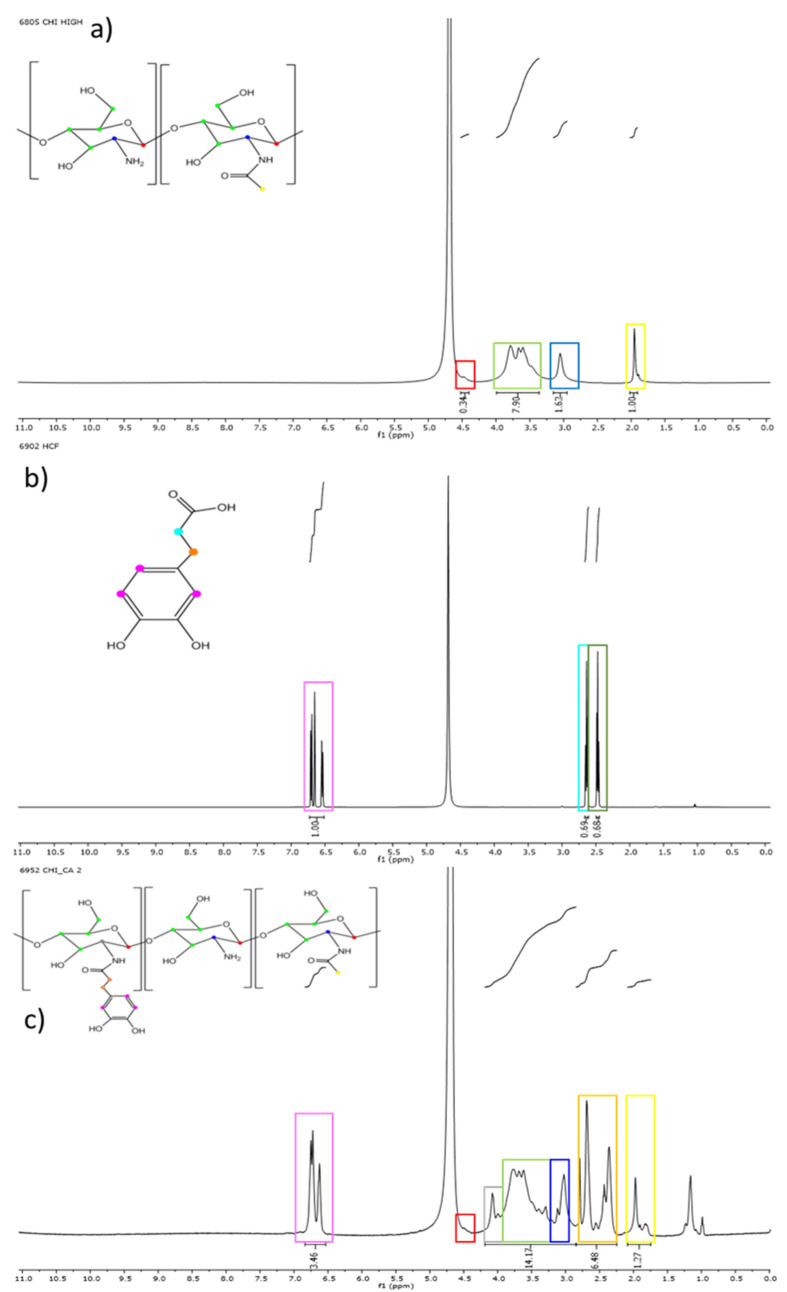
(**a**) High molecular weight chitosan spectrum. (**b**) Hydrocaffeic acid spectrum. (**c**) Chitosan modified with catechol (CHI-CA) spectrum.

In the CH-CA spectrum (Figure 7c), the appearance of the characteristic peaks of catechol groups (6.5–6.75 ppm) can be observed, indicating that the reaction takes place successfully. In addition, the peaks at 2.5 ppm corresponding to the aliphatic carbons of hydrocaffeic acid and the appearance of the signal at 4 ppm, which corresponds to the hydrogen of the C2 of the glucosamine unit of the chitosan bound to catechol, were also observed. The percentage of substitution of catechol was calculated by the integration of the peak at 6.5–6.75 ppm with respect to that of chitosan appearing at 1.99 ppm, knowing the degree of deacetylation. Different synthesis conditions were explored in CHI modification. On the one hand, the following results were obtained: CHI-CA 1 with 24 h of reaction and 1:2 CHI:HCF feed ratio, CHI-CA 2 with 8 h of reaction and 1:2 CHI:HCF feed ratio and, finally, CHI-CA 3 with 12 h of reaction and 1:1 CHI:HC feed ratio. The resulting percentage of catechol varied according to these synthetic conditions (Table 1). Indeed, lower reagent equivalents (CHI-CA 3) and reaction time (CHI-CA 2) resulted in a significant decrease in the conjugation with catechol.

1H-NMR Chitosan (D2O, 500 MHz, 20 °C): δ (ppm) = 4.50 (s, 2H, anomeric CH), 3.30–4.00 (m, 10H, ring CH and CH2), 3.10 (s, 2H, CH -N ring), 1.99 (s, 3H, acetamide).

1H-NMR Hydrocaffeic acid (D2O, 500 MHz, 20 °C): δ (ppm) = 6.5–6.75 (m, 3H, CH of the aromatic ring), 2.60 (d, 2H, CH2COOH), 2.50 (d, 2H, CH2Ar).

1H-NMR Chitosan-catechol (D2O, 500 MHz, 20 °C): δ (ppm) = 6.5–6.75 (m, 3H, CH of the aromatic ring), 4.50 (s, 3H, anomeric CH), 4.20 (dd, 1H, CH-N (catechol)), 3.25–3.80 (m, 15H, ring CH and CH2), 3.10 (s, 2H, CH-N), 2.60 (d, 2H, CH2COOH), 2.30 (d, 2H, CH2Ar), 1.99 (s, 3H, acetamide).

### 3.2. Hydrogel Formation of Catechol Derivatives

The spontaneous oxidation of the catechol group leads to their transformation to the quinone group that presents an absorption in the visible spectrum at λ = 380–480 nm (depending on the degree of oxidation) [27]. Accordingly, the quantification of quinone moiety was carried out by using the calibration curve obtained with a standard solution of 1 mM dopamine hydrochloride previously oxidized with sodium periodate (1:1 Dopa/Periodate). When periodate was added to the dopamine solution, it immediately took on a yellow hue, and after 10 min, it became reddish and brown, since the absorption spectrum varies with oxidation time. For this reason, the calibration was carried out at the isosbestic point, at which absorption is not a function of time [27] (λ = 413.6 nm, Abs = 1112C + 0.009R^2^ = 0.991). This color change allows monitoring the oxidation of catechol-modified polysaccharides.

**Figure 8:**
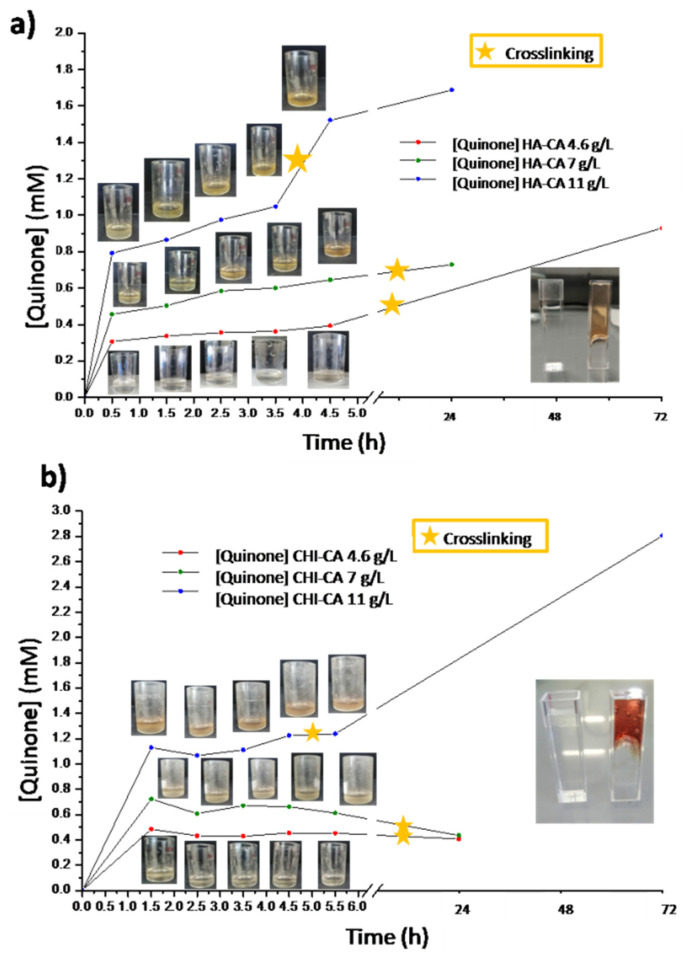
Quinone concentration over the time in (**a**) HA-CA and (**b**) CHI-CA samples calculated from calibration. In addition, a yellow star indicates the gelation time determined by the vial inversion method.

**Figure 9:**
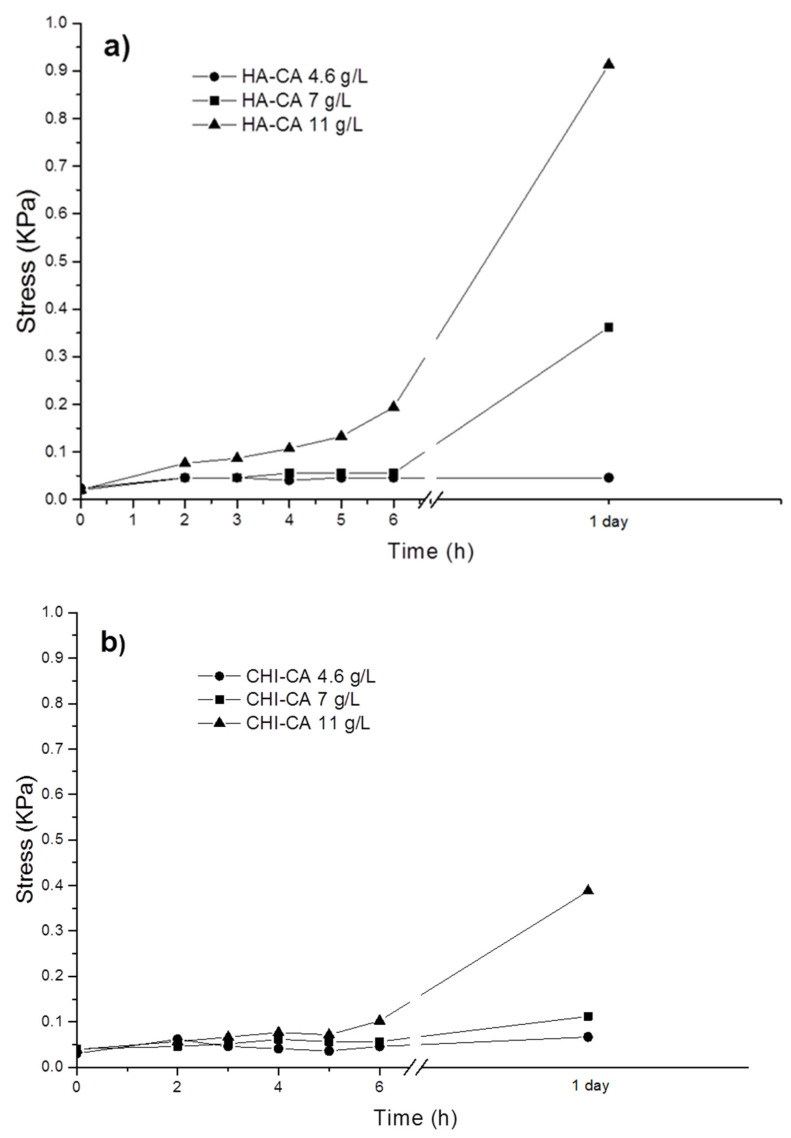
Skin adhesion graph of (**a**) HA-CA and (**b**) CHI-CA over time at different concentrations.

It is known that this oxidation of catecholized polymers solution with air [9,12,23] causes sol–gel transition that changes adhesion to skin. Taking all this into account, the oxidation of HA-CA and CHI-CA was analyzed along the time for different polymer concentrations in terms of the variation of tissue adhesion and quinone concentration (Figure 8 and Figure 9). As expected, as the time of exposure to air increases, as a consequence of the appearance of the oxidized species, o-quinone, the increase in coloration took place (Figure 8) and the characteristic absorption band at 413.6 nm was observed for both polymers. It is also shown that higher concentration of catechol-derived polymer in the solution leads to a greater quinone concentration. For instance, in the case of 11 g/L of HA-CA, the presence of quinone during the first 30 min is four times higher than that of 4.6 g/L solution. In addition, an increase in viscosity was observed, caused by the gelling of the polymers through the formation of covalent bonds between quinones, originating a covalent three-dimensional network that results in the formation of the hydrogel [22]. This spontaneous formation of the gels can be considered a great advantage since, as mentioned above, it allows obtaining cross-linked systems without additional reactions or reagents. The gelation time, determined by the vial inversion method [9], is indicated in Figure 8 (yellow star) for all studied concentrations.

Regarding CHI-CA, it is worth highlighting that the quinone concentration is higher than that for HA-CA for all studied concentrations. This difference can be ascribed to the higher degree of catechol substitution in the CHI-CA (82%) in comparison with HA-CA (38%). Tissue adhesion tests were also carried out at different modified polysaccharide concentrations (4.6, 7, 11 g/L). The stress–displacement curves were obtained, and the maximum stress points required for tissue-polymer detachment were analyzed (Figure 9).

It could be observed (Figure 9) for both polymers that as the concentration of initial catechol groups and oxidation time increases, where quinone content increases, a greater detachment force is measured. As it is known, in organic surfaces such as porcine skin, which contains thiol and amino groups [12], quinone is more adhesive than catechol [17] due to the covalent nature of the formed quinone bonds compared with the weaker physical interactions established by catechol groups [9,12,21]. When HA-CA and CHI-CA are compared, it can be highlighted that a higher adhesion was measured for HA-CA than CHI-CA samples, despite the higher catechol content of CHI-CA samples. This is because CHI-CA undergoes faster and additional self-crosslinking [12]. Moreover, in addition to intramolecular reactions between quinones, the amine group from the deacetylated CHI segments also reacts through Michael additions with quinone [22]. Thus, there is a lower content of quinone moieties available to tissue interaction than in the case of the HA-CA system. Additionally, hydration is a key factor in adhesion, as it enhances the mobility of the polymer chains that promotes tissue adhesion [28]. In this sense, HA is one of the most hydrating polymers known [29,30], which can interestingly enhance the tissue adhesion of HA-CA gels.

**Figure 10:**
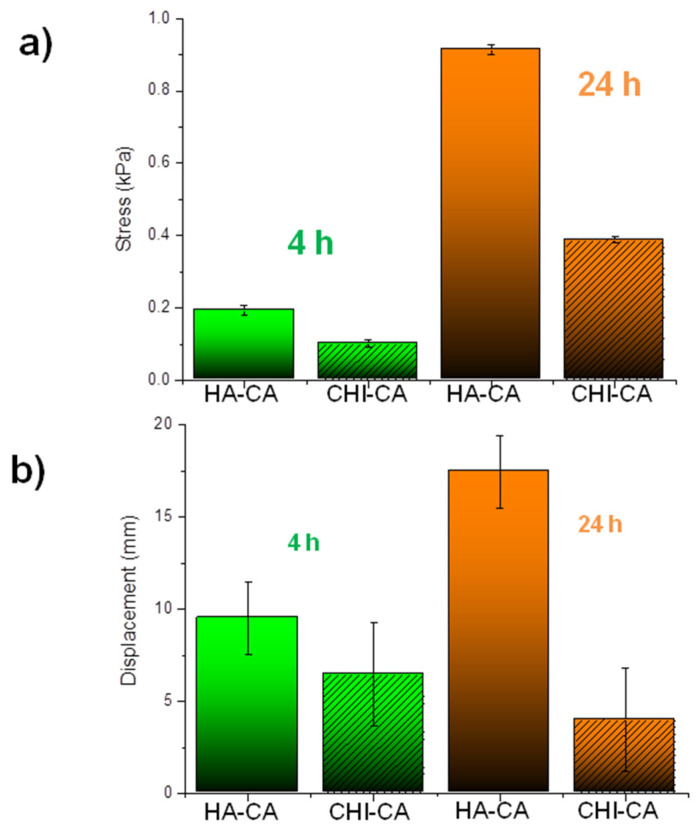
Comparative graph between the studied systems that gathers (**a**) the maximum stress applied to detach the sample from the skin and (**b**) the maximum elongations until rupture of the systems studied after 4 h (green) and after 24 h (orange) for HA-CA and CHI-CA hydrogels. *n* = 5.

Figure 10 shows specifically the stress applied at the detachment (Figure 10a) and the displacement produced by the gels before breakage (Figure 10b) for both catechol modified polysaccharides after 4 and 24 h of oxidation by air exposure. As it is observed in the stress graphs (Figure 10a), the adhesion of HA-CA is greater than that of CHI-CA even though a greater content of quinone was determined by VIS spectroscopy.

As it is observed in the stress graphs (Figure 10a), the adhesion of HA-CA is greater than that of CHI-CA even though there is a greater content of quinone determined by VIS spectroscopy. In addition to this, it is known that CHI hydrates more slowly than HA [29,31], which has a negative influence on adhesion, as we have already mentioned. Indeed, hydration, which has the function of a lubricant, seems to increases the mobility of HA chains in contrast to CHI, which improves adhesion properties.

### 3.3. Microscopy Analysis of the Film

**Figure 11:**
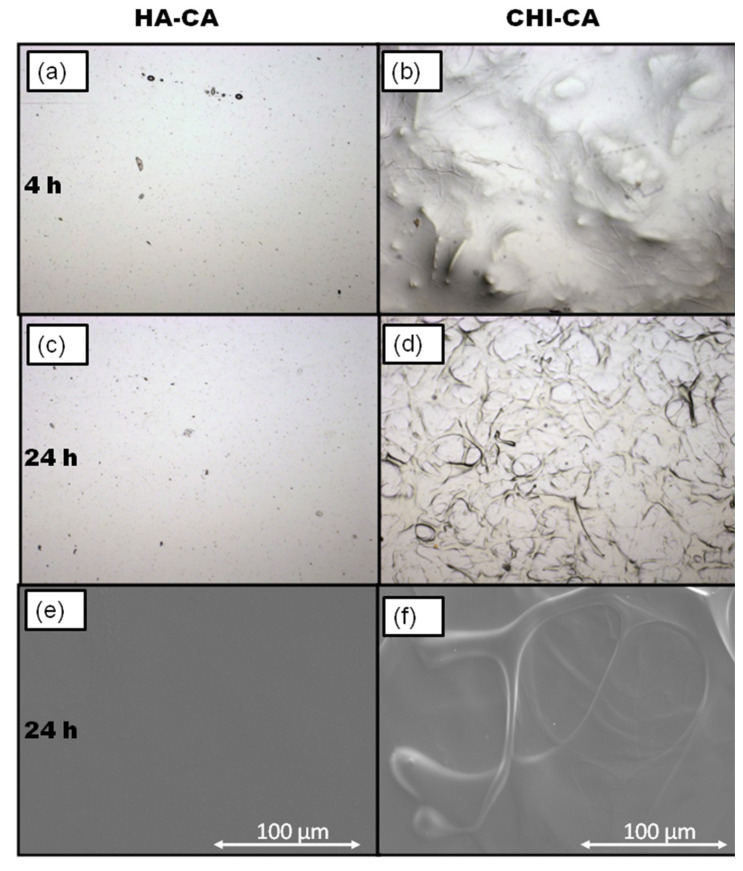
(**a**) HA-CA photographs taken with microscope after 4 h with (×4 lens and 1.62 µm/pixel) and (**b**) CHI-CA photographs taken with microscope after 4 h with (×4 lens and 1.62 µm/pixel) (**c**) HA-CA photographs taken with microscope after 24 h (×4 lens and 1.62 µm/pixel) (**d**) CHI-CA photographs taken with microscope after 24 h of drying with ×4 lens (1.62 µm/pixel) (**e**) HA-CA SEM images after 24 h of drying and (**f**) CHI-CA SEM images after 24 h of drying.

The filmogenic ability of wound dressing materials is a valuable property due to the fact that it promotes wound protection and reduces bacterial growth [14]. For this reason, the films of the studied systems were developed by casting CA-modified polysaccharide solution, as described in the experimental section. After 4 h (Figure 11a,b) and 24 h (Figure 11c,d) of drying, photographs were taken under the light microscope to HA-CA and CHI-CA hydrogel films in order to study the possible formation of fractures and the homogeneity of the films as a non-destructive technique. High resolution images were also obtained by scanning electron microscopy (SEM) for HA-CA (Figure 11e) and CHI-CA (Figure 11f) after 24 h of drying for more information on the micron scale [32].

After 4 h of drying, noticeable differences between some systems can be appreciated. In the case of HA-CA films (Figure 11a), photographs indicate high homogeneity of the film without cracks or wrinkles. However, for CHI-CA films (Figure 11b), high roughness can be observed, probably due to lower solubility in the water of polymers. In addition, its poor filmogenic capacity is observed as a consequence of its high drop forming ability during the casting process that is indicative of the existence of important cohesive forces [32], which corresponds to those derived from highly crosslinked CHI-CA (quinone-NH2) [12,22].

After 24 h of drying, the HA-CA hydrogels do not display difference with 4 h of drying (Figure 11c), while signs of increased crosslinking were observed as roughness or irregularities due to the formation of small aggregates in the case of CHI-CA. Indeed, CHI-CA (Figure 11d) shows fiber-like structures as a consequence of solvent evaporation from its previous gelled aggregates. The rapid crosslinking of CHI-CA seems to decrease the homogeneity of the film, and high cohesion forces do not allow smooth surface formation.

Finally, SEM images taken after 24 h of drying confirmed that highly stable films are obtained for HA-CA gels (Figure 11e). However, regarding CHI-CA (Figure 11f), fibers remained after the total elimination of the solvent in high resolution due to its poor ability to spread, high cohesion and crosslinking forces [22].

## 4. Conclusions

The conjugation of CHI and HA with catechol groups was successfully developed and quantified by ^1^H-NMR spectroscopy. The spontaneous oxidation of introduced groups to o-quinone by the action of air resulted in hydrogel formation that could be easily observed by the change of color along the polymeric chains where crosslinking took place. Higher catechol content, derived from higher polymer concentration as well as higher modification degree, resulted in greater quinone concentrations that resulted in faster gelation for both polymers. In addition, it was demonstrated that gelation induces a clear variation on tissue adhesiveness of these catecholized polymers. As HA-CA and CHI-CA air induced gelation, an increase in tissue detachment force was measured in stress–strain curves due to the stronger interactions of quinone groups with tissue in comparison with initial catechol moiety. CHI-CA spontaneous hydrogels showed reduced adhesiveness in comparison with HA-CA, even though its initial catechol content was greater higher due to the additional reaction of quinone groups with free amine groups present along CHI polymer. In addition, the analysis of the comparative filmogenic ability of CHI-CA and HA-CA points out a lower homogeneity of CHI-CA, possibly ascribed to the lower solubility of this conjugate, as well as for its additional crosslinking and consequent viscosity. The spontaneous gelation of HA-CA and CHI-CA allows obtaining highly cross-linked systems without additional reactions or reagents, which in the case of HA-CA interestingly show also excellent filmogenic properties and n enhanced tissue-adhesive ability.

## Figures and Tables

**Table 1:** Substitution percentages of catechol in the samples.

Sample	Catechol % (^1^H NMR) ^a^
HA-CA	38 ± 8
CHI-CA 1	82 ± 10
CHI-CA 2	8 ± 2
CHI-CA 3	2 ± 2

^a^*n* = 3.

## Data Availability

The data presented in this study are available upon request from the corresponding author.
